# White matter alterations in first episode treatment-naïve patients with deficit schizophrenia: a combined VBM and DTI study

**DOI:** 10.1038/srep12994

**Published:** 2015-08-10

**Authors:** Wei Lei, Na Li, Wei Deng, Mingli Li, Chaohua Huang, Xiaohong Ma, Qiang Wang, Wanjun Guo, Yinfei Li, Lijun Jiang, Yi Zhou, Xun Hu, Grainne Mary McAlonan, Tao Li

**Affiliations:** 1 The Mental Health Center & Psychiatric Laboratory, State Key Laboratory of Biotherapy, West China Hospital, Sichuan University, Chengdu City, Sichuan Province, China.; 2 Department of Radiology, Hospital of Chengdu Office of People’s Government of Tibetan autonomous Region, Branch Hospital of West China Hospital, Sichuan University, Chengdu City, Sichuan Province, China.; 3 Huaxi Biobank, West China Hospital, Sichuan University, Chengdu City, Sichuan Province, China.; 4 Department of Forensic and Neurodevelopmental Sciences, Institute of Psychiatry, Psychology and Neuroscience, King’s College, London, UK.

## Abstract

Categorizing ‘deficit schizophrenia’ (DS) as distinct from ‘non-deficit’ schizophrenia (NDS) may help reduce heterogeneity within schizophrenia. However, it is unknown if DS has a discrete white matter signature. Here we used MRI to compare white matter volume (voxel-based morphometry) and microstructural integrity (fractional anisotropy, FA) in first-episode treatment-naïve patients with DS and NDS and their unaffected relatives to control groups of similar age. We found that white matter disruption was prominent in DS compared to controls; the DS group had lower volumes in the cerebellum, bilateral extra-nuclear and bilateral frontoparietal regions, and lower FA in the body of corpus callosum, posterior superior longitudinal fasciculus and uncinate fasciculus. The DS group also had lower volume in bilateral extra-nuclear regions compared to NDS, and the volume of these clusters was negatively correlated with deficit symptom ratings. NDS patients however, had no significant volume alterations and limited disruption of microstructural integrity compared to controls. Finally, first-degree relatives of those with DS shared volume abnormalities in right extra-nuclear white matter. Thus, white matter pathology in schizophrenia is most evident in the deficit condition, and lower extra-nuclear white matter volumes in both DS patients and their relatives may represent a brain structural ‘endophenotype’ for DS.

Clinical and genetic heterogeneity within schizophrenia has contributed to a relative lack of progress in understanding the illness^1^,^2^. The concept of deficit schizophrenia (DS) is regarded as one of the most promising attempts to reduce the heterogeneity within the spectrum^3^. It is proposed that those with DS represent a clinically homogeneous subgroup of patients with primary and enduring negative symptoms^4^. Consistent with this, the diagnosis of DS, separate from non-deficit schizophrenia (NDS), has been demonstrated as stable and enduring^5^,^6^. Compared to NDS, DS is characterized by more impaired social, global cognition and executive functions, and worse long-term prognosis. The neurobiological underpinnings of DS and NDS have also been suggested to be at least partly distinct^7^.

Clinically homogeneous subgroups within spectrum conditions such as schizophrenia may have a recognizable ‘endophenotype’, a biological or behavioral marker thought to reflect an inherited vulnerability for the disorder^8^. Endophenotypes are state-independent, co-segregate within families, and are found in some unaffected relatives of individuals with the disorder (though to a milder extent than in patients)^9^. Identifying endophenotypes may be a beneficial strategy in investigations of complex and multi-factorial, inherited disorders such as schizophrenia^10^. The rationale being that the number of genes involved in a phenotype is directly related to the complexity of the phenotype^11^.

In support of this, there is evidence that, compared to the relatives of those with NDS, the family members of individuals with DS tend to have a higher prevalence of subclinical deficit symptoms, such as social withdrawal; they also have an increased risk of schizophrenia^12^,^13^. However, no study has extended the investigation of brain biology in DS and NDS to include family members.

Cerebral white matter subserves all cognitive and neurological functions through fiber pathways which link geographically distant cortical and subcortical regions^14^. White matter abnormalities have therefore been associated with a number of psychiatric and affective manifestations^15^,^16^. For example, lower white volumes have been reported across whole brain^17^, corpus callosum^18^, bilateral frontal lobe and internal capsule^19^ in schizophrenia and white matter pathology has been linked to both negative and positive symptoms^16^. In addition, DTI studies of schizophrenia indicate that lower FA measures in fronto-limbic-striatal circuits^20^,^21^.

Such white matter anomalies are particularly prominent in studies of individuals with chronic DS^22^,^23^,^24^,^25^,^26^,^27^,^28^. For example, DS is associated with greater density of interstitial cells of the white matter in frontal and parietal lobe^26^,^27^ and disrupted fiber integrity in superior longitudinal fasciculus and uncinate fasciculus^22^,^24^,^25^. Thus the pattern of white matter pathology in DS may constitute an ‘endophenotypic’ marker. However, in imaging studies, the range of techniques used, small sample sizes, heterogeneous treatment conditions, or duration of illness, may confound interpretation of DS studies^3^; and to examine potential white matter ‘endophenotypic’ markers of DS, a study of untreated first-episode patients is needed, alongside a study of their relatives.

Therefore, we conducted a bimodal imaging analysis to compare both white matter volume (voxel-based morphometry, VBM) and microstructural integrity (fractional anisotropy, FA) in first-episode and treatment-naïve patients with schizophrenia, with either DS or NDS, their unaffected first-degree relatives and matched healthy controls. Symptom severity of patients was evaluated using the Positive and Negative Syndrome Scale (PANSS)^29^. We addressed the following questions: (1) Does DS have distinct patterns of white matter alterations at first clinical presentation relative to controls and those with NDS? (2) If so, are these linked to negative symptom severity? Finally, (3) are similar white matter structural differences also present in their non-psychotic relatives?

## Results

### Demographic and clinical characteristics

The demographic and clinical characteristics of the participants are shown in Table 1. Compared to respective control groups, patients and relatives showed lower IQ, there were no statistically significant differences in whole brain volume, total white matter volume, age, gender, and educational years in patients or relative groups. However, as expected DS patients had longer duration of untreated psychosis (DUP), higher PANSS total scores and PANSS negative subscale scores than NDS patients. There was no significant difference between two patient groups at age of illness onset, PANSS positive subscale scores and PANSS general psychopathological subscale scores.

### Voxel-wise comparisons of white matter volume

Results of voxel-wise comparisons are shown in Table 2.

#### Patients

**Figure 1:**
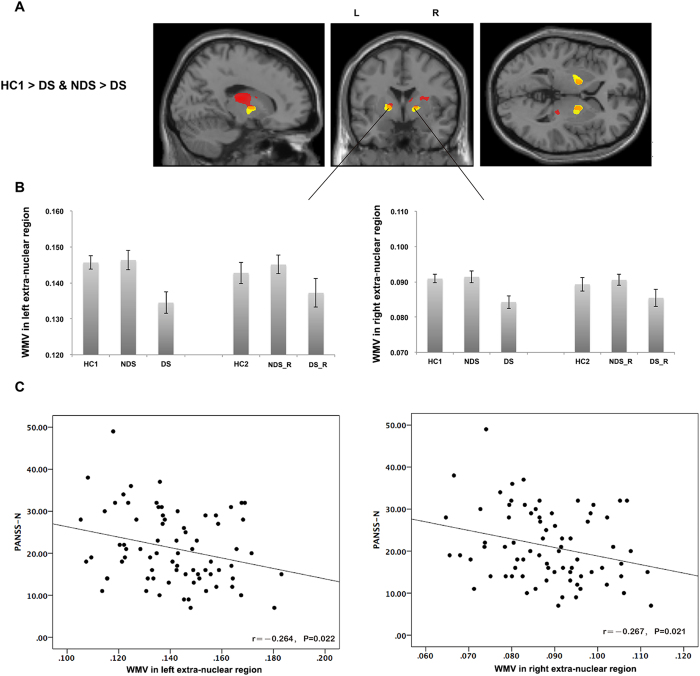
Volume lose in extra-nuclear white matter in DS patients. DS patients had lower white matter volume in bilateral extra-nuclear regions compared to both healthy controls (red) and NDS patients (yellow) (**A**,**B**). The white matter volume of bilateral extra-nuclear regions (the overlapping regions) was inversely correlated with PANSS negative subscale scores in patients (**C**). *Note: L, Left hemisphere; R, right hemisphere; WMV, white matter volume.*

Compared with matched healthy controls (HC1), DS patients had significantly lower white matter volume in the posterior lobe of the cerebellum and bilateral extra-nuclear regions (around the basal ganglia, extending to insula); and greater white matter volume in bilateral sub-lobar frontoparietal areas. In contrast, NDS patients had no significant white matter volume alterations. Direct comparison of DS and NDS patients revealed that the volume of extra-nuclear white matter was smaller in DS patients (Fig. 1A,B).

#### Relatives

**Figure 2:**
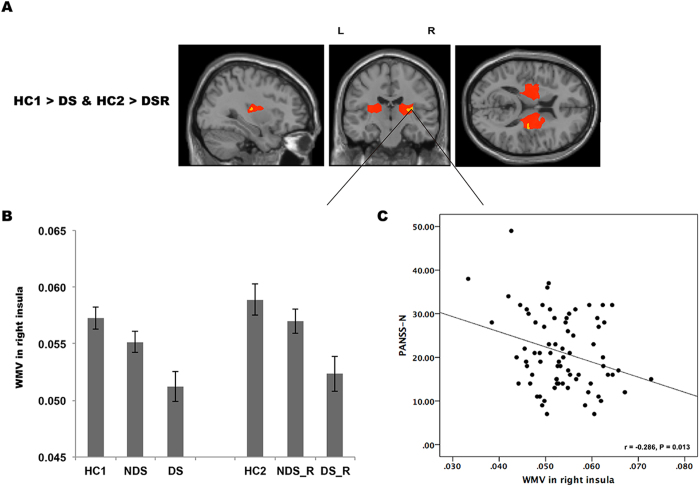
A potential endophenotype of DS. Both the DS patients (red) and their relatives (yellow) had lower white matter volume in sub-lobar of right insula compared with corresponding controls (**A**,**B**). The white matter volume of right insula was inversely correlated with PANSS negative subscale scores in the combined patient groups (**C**).

Compared with healthy controls (HC2), relatives of DS patients had significantly less white matter volume in the right insula. This overlapped with the right extra-nuclear cluster where the DS probands had less volume than HC1 (Fig. 2A,B). Relatives of DS patients also had significantly smaller white matter volume in bilateral extra-nuclear regions compared to relatives of NDS patients. No significant volume abnormalities were evident in relatives of NDS patients.

### Voxel-wise comparisons of FA

#### Patients

**Figure 3:**
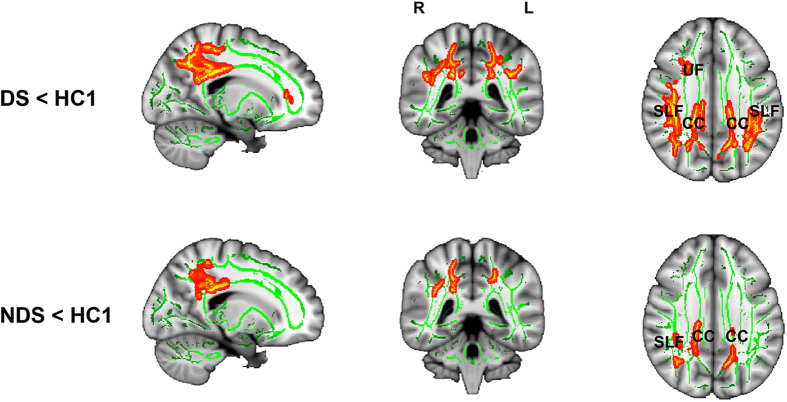
Fractional anisotropy (FA) differences in DS patients (upper panel) and NDS patients (bottom panel). The regions showing lower FA in patients compared with healthy controls are shown in red-yellow. *Note: SLF, Superior Longitudinal Fasciculus; CC, corpus callosum. UF, uncinate fasciculus.*

Relative to controls, FA was significantly lower in the body of corpus callosum and the posterior superior longitudinal fasciculus bilaterally in both DS and NDS groups; but regions with lower FA extended to include the right uncinate fasciculus in the DS group (Table 3 and Fig. 3). Direct comparison of whole brain FA maps from DS and NDS groups did not reveal any differences; However, the mean FA extracted from the uncinate fasciculus ROI (which showed an FA difference between patient groups and controls) was significantly lower in the DS group than the NDS group (t_73_ = 2.852, p = 0.006).

#### Relatives

No significant FA differences were found in comparisons including either relative group, even when a threshold of uncorrected p < 0.01 was applied.

### Region of interest analyses

#### Correlations with symptoms

The bilateral extra-nuclear and right insula clusters were defined as regions of interest (ROIs) for subsequent correlation analysis. There was a significant inverse correlation between negative symptoms and white matter volume of the left (r = −0.264, p = 0.022) and right (r = −0.267, p = 0.021) extra-nuclear clusters; and significant inverse correlation right insula (r = −0.286, p = 0.013) across combined patient groups (Figs 1C and 2C). There were no significant correlations between IQ (or DUP) and white matter volumes of the ROIs.

#### The relationship between volume and microstructure (FA) alterations in white matter

No group differences in FA were detected in any of the ROIs with significant group volume differences.

### Post hoc analysis for potential confounders

The DUP was longer in DS patients than NDS patients in our sample. To rule out potentially confounding effects of DUP on the group differences in volume observed, two post hoc analyses were carried out in SPM8. First, a regression analyses using DUP as predictor was performed within the white matter volume maps. As the DUP data was log-transformed to conform to assumptions of normality (Kolmogorov-Smirnov Z = 0.998, p = 0.272). Next, a median split of DUP data was used to divide the NDS group into two groups (each n = 21) with shorter and longer DUP and their white matter indices were compared. None of these analyses revealed a significant effect of DUP on white matter volume of extra-nuclear, even at a tolerant threshold of uncorrected p < 0.01(see Supplementary Information). Thus differences in DUP were unlikely to account for white matter volume differences between DS and NDS groups.

## Discussion

In present study, we identified a distinct pattern of white matter alterations in DS patients compared to both healthy controls and NDS patients. We found that, relative to healthy controls, patients with DS (but not NDS) had white matter volume alterations in the cerebellum, bilateral extra-nuclear and bilateral frontoparietal regions. Furthermore, patients with DS had smaller white matter volumes in bilateral extra-nuclear regions compared to those with NDS. We also found that the first-degree relatives of patients with DS shared white matter volume abnormalities in the right insula (a part of the extra-nuclear cluster) with their probands. Greater regional volume deficits in bilateral extra-nuclear were associated with more severe negative symptoms across both patient groups. In addition, compared to controls, patients with DS and NDS had lower FA in the body of corpus callosum and posterior superior longitudinal fasciculus; however only patients with DS had lower FA in the uncinate fasciculus.

In this study, we observed white matter volume alterations in a DS group that correspond closely to results of previous studies of more heterogeneous groups of individuals with first-episode schizophrenia. Previous anatomical studies of schizophrenia have reported differences in the frontoparietal^30^, extra-nuclear^31^,^32^ and cerebellar^33^ regions. Of these, volume alterations in fronto-limbic white matter is one of the most consistent findings [see meta-analyses^19^,^21^].

In contrast, differences in FA between DS and NDS groups were less prominent. Compared to controls, both patient groups had lower FA in bilateral corpus callosum and superior longitudinal fasciculus, and left cingulum. This pattern is consistent with previous studies of first-episode patients^34^,^35^,^36^. However, differences between patients with DS and controls extended to the uncinate fasciculus; and this pattern is similar to the abnormalities reported to accompany chronic DS^22^,^24^,^25^. Our ROI comparison of uncinate fasciculus FA measures in the patient groups confirmed that FA was lower in this region in DS. Taken together, these results suggest that there are some common microstructural white matter alterations in fronto-limbic circuitry in DS and NDS in the early stages of the disorder, but pathology in DS is more extensive than NDS.

Our study reveals that a relatively selective volume reduction in bilateral extra-nuclear pathways characterizes DS; and that there are similar abnormalities in first-degree relatives of those with DS. These results fit with previous reports of volume deficits in extra-nuclear white matter and in the superior longitudinal fasciculus in patients with primary negative symptoms^23^, or severe negative symptoms^37^. Disrupted white matter connections between the prefrontal cortex and other cortical regions such as the temporal, insular and occipital cortices; and to sub-cortical structures such as the striatum have been consistently linked with the severity of negative symptoms^25^,^38^. Nevertheless, the exact role extra-nuclear white matter in DS is not clear. This extra-nuclear region contains major fibers linking the striatum and frontoparietal cortex, and structural alterations in this pathway could potentially affect the cortico-striatal mechanisms of reward^39^. In addition, functional abnormalities within the cortico-striatal system are associated with two major negative symptoms, amotivation and anticipatory anhedonia^40^,^41^. Thus, structural alterations in the pathway observed here could provide a basis for this functional – and subsequently clinical – phenotype.

Lower extra-nuclear white matter volume is also thought to contribute to the ventricular enlargement^31^ associated with DS and with features such as poor response to treatment, poor outcome and greater negative symptoms^42^,^43^. We captured a similar ‘pathology’ in relatives of patients with DS, suggesting that this volume deficit may be genetically determined. That is, these findings may reflect neurodevelopmental abnormalities which disrupt neuronal migration and organization^44^ in those at increased genetic risk^45^,^46^. This fits with the proposal that DS might represent an early onset non-progressive developmental process which interferes with the acquisition of basic cognitive and social skills from childhood^3^. It is also consistent with evidence that patients with DS have poor premorbid adjustment across all stages of development; impairment in those with NDS tends to appear in late adolescence or early adulthood^47^.

White matter volume and FA abnormalities in DS were observed in different brain regions. This may indicate that discrete mechanisms disrupt macrostructural volume and microstructural fibre indices early in the course of schizophrenia. This explanation fits with results from prior studies reporting a similar ‘dissociation’ between white matter volume and FA in patients with schizophrenia^48^,^49^. The underlying reason for this dissociation is not clear and may be multi-factorial. For example, lower FA with normal volume measures could arise in regions where there is fiber crossing, myelin loss, a change in the chemical composition of the white matter or differences in the proportion of axonal, oligodendrocyte and glial cellular components^20^,^50^. Further research will however be needed to better understand the cellular underpinnings of white matter abnormalities in schizophrenia.

## Limitations

There are a number of limitations to our study. 1) The main clinical difference between our DS and NDS groups was a longer DUP in DS group. To address this, we conducted post hoc analyses and the results indicated that the extra-nuclear white matter volume differences in DS were independent of DUP. 2) We used PANSS scores and IQ to explore the clinical and cognitive significance of white matter changes. However, further more specific psychological or cognitive assessment targeted at the deficit syndrome would help us better understand the functional meaning of MRI findings. This will be a focus of future studies. 3) The 15 diffusion gradients DTI data used in this study could also have limited our sensitivity to detected changes in microstructural integrity of white matter fibres. However, at the start of the study we used what was optimal at the time and during the course of this large study we elected not to change the scanning parameters. 4) Finally, as in any study of schizophrenia there are multiple potential confounds such as lifestyle, diet, exercise, relatively low-dosage alcohol and tobacco usage. These are acknowledged as potentially important as they may independently impact on brain structure^51^,^52^.

In this study, we identified a distinctive pattern of white matter alterations in first-episode patients with DS. Specifically, we propose that lower volume in extra-nuclear white matter may be a biological marker of DS. To our knowledge, this is the first report of a neuroanatomical endophenotype for a subgroup of schizophrenia. The results should encourage further study of clinically and biologically homogenous samples within the schizophrenia spectrum.

## Methods

### Participants

Two hundred and eleven subjects, including 75 first-episode patients (33 DS and 42 NDS) with schizophrenia, 58 unaffected first-degree relatives of these patients, and 78 healthy controls participated in this study.

All patients were recruited at the Mental Health Centre of the West China Hospital, Sichuan University. Diagnoses of schizophrenia were confirmed using the Structured Clinical Interview for the DSM-IV Patient Edition (SCID-I/P)^53^ shortly after presentation to the mental health services in their first episode of psychotic illness. Data collected from patients initially diagnosed with schizophreniform psychosis (n = 20, 6 DS and 14 NDS) were included if a diagnosis of schizophrenia was confirmed after at least 6 months follow-up. The diagnoses of DS and NDS were reached using the Schedule for the Deficit Syndrome (SDS)^54^, 12 months after admission.

The psychiatric history of each patient was reviewed in order to exclude patients with a history of any other major psychiatric disorder (including schizophrenia, bipolar disorder, depression and schizoaffective disorders), mental retardation, head trauma, current substance abuse and neurological disorders. Information about previous treatment and duration of DUP was collected at diagnosis. Five patients (6.6%; 2 DS and 3 NDS) were taking low dose antipsychotics (risperidone or olanzapine; 25 to 75 mg of chlorpromazine daily dose equivalent) for less than 3 days prior to MRI. All others (93.4%, 23 DS and 39 NDS) were treatment-naïve before scanning. Symptom severity was evaluated using the Positive and Negative Syndrome Scale (PANSS) in the patient groups^29^.

Current IQ was estimated for all subjects using the seven-subtest version (including Information, Similarities, Arithmetic, Digit Span, Picture Completion, Block Design, and Digit Symbol subtests)^55^ of the Wechsler Adult Intelligence Scale-revised in China (WAIS-RC)^56^.

All controls and relatives were screened for a lifetime absence of Axis I illness of DSM-IV psychiatric illnesses with the SCID non-patient version (SCID-I/NP)^57^. Relatives were assigned to two subgroups; relatives of DS (DS_R) or relatives of NDS (NDS_R). Because relatives were generally older than first-episode patients, two groups of ‘healthy’ controls were age- and sex-matched with patients (HC1) and relatives (HC2) to avoid confound the brain structural differences due to age^58^. Relatives or controls with any major psychiatric or neurological disorder, serious physical illness, alcohol or drug abuse, pregnancy, head trauma, or mental retardation were excluded from the study. We excluded subjects with a history of alcohol abuse, according to DSM-IV criteria. The number of smokers in HC1, NDS and DS groups was 5, 10 and 8, respectively. The number of smokers in HC2, NDS_R and DS_R was 8, 12 and 8 respectively. The proportion of smokers was not significantly different across patient groups (χ^2^ = 2.001, p = 0.368), or relative groups (χ^2^ = 1.353, p = 0.508).

All participants were Han Chinese and right-handed as assessed by the Annett Handedness Scale^59^. Written informed consent was obtained from all participants individually, and with their guardians as appropriate. This study was carried out in accordance with the Declaration of Helsinki and was approved by were approved by the Institutional Review Board of West China Hospital, Sichuan University.

### MRI data acquisition

#### Structural MRI

High-resolution T1-weighted images were acquired from patients on admission using a 3-Tesla MRI system (EXCITE, General Electric, WI, USA) and an eight-channel phase array head coil with a volumetric 3D Spoiled Gradient Recall (SPGR) sequence (TR = 8.5 ms, TE = 3.4 ms, slice thickness = 1.0 mm with no gap, field of view = 24 × 24 cm^2^, matrix size = 256 × 256), generating 156 contiguous axial slices with the in-plane resolution of 0.47 mm × 0.47 mm.

#### DTI

Whole brain diffusion-weighted images were recorded along 15 gradient directions (b = 1000 s/mm^2^, number of excitations = 2) together with one unweighted (b = 0) image (42 images in total). Each image was acquired using a single-shot spin echo planar imaging (EPI) sequence (TR = 10 000 ms, TE = 70.8 ms, slice thickness = 3.0 mm with no gap, field of view = 24 × 24 cm^2^, matrix size = 128 × 128, voxel resolution 0.94 × 0.94 × 3 mm^3^).

An experienced neuroradiologist qualitatively inspected the raw MRI data. No gross abnormalities were observed for any participant.

### MRI data analysis

#### Structural MRI

Structural data were analyzed using SPM8 (http://www.fil.ion.ucl.ac.uk/spm) running on Matlab7.6 (MathWorks, Inc., Natick, MA, USA). The preprocessing included the following steps: (1) T1-weighted images were realigned and then manually reoriented so that the anterior commissure was positioned at coordinate [0, 0, 0]; (2) MINC non-parametric, non-uniformity intensity normalization software (http://wiki.bic.mni.mcgill.ca /index.php/MINC) was used to rectify the uniformity of high-magnetic field signals in reoriented images; (3) the images were then segmented into gray matter, white matter, and cerebrospinal fluid probability maps using the unified segmentation option; (4) The white matter images were spatially normalized to MNI space and Jacobian modulated into volume images using the Dartel (Diffeomorphic Anatomical Registration using the Exponentiated Lie algebra) toolbox^60^ implemented in SPM8, and finally (5) smoothed with a 6 mm FWHM Gaussian kernel.

#### DTI

DTI data were preprocessed using the FMRIB’s Diffusion Toolbox (FDT) within FSL (http://www.fmrib.ox.ac.uk/fsl). The preprocessing included the following steps: (1) the diffusion-weighted volumes were aligned to their corresponding b0 image with an affine transformation to minimize image distortion from eddy currents and head motion. (2) The non-brain tissue and background noise were removed from b0 image using BET. (3) The diffusion tensor for each voxel was estimated by the multivariate linear fitting algorithm. Finally (4) voxel-wise values of FA (fractional anisotropy) were calculated.

Whole brain analysis of FA images was performed using the TBSS toolbox^61^. In brief, FA maps of all subjects were first realigned to a common target and then normalized to a 1 × 1 × 1 mm^3^ Montreal Neurological Institute standard space (MNI152) via the FMRIB58_FA template. Thereafter, the registered FA images were averaged to generate a cross-subject mean FA image, and the mean FA image applied to create a mean FA skeleton representing the center of all fiber tracts common to the group. The mean FA skeleton was further thresholded by a FA value of 0.2 to exclude peripheral tracts with significant inter-subject variability and/or partial volume effects due to grey matter. Each participant’s aligned FA dataset was then projected onto this skeleton.

### Statistical analysis

#### Demographic data

Statistical analysis of demographic data was conducted using SPSS16.0 for Windows (SPSS Inc., Chicago, IL, USA). Where appropriate parametric (analysis of variance, ANOVA: or Student’s t-test) and non-parametric (Mann-Whitney’s U test) procedures were used to compare the distribution and differences of categorical and continuous data between groups. For categorical data, χ^2^ tests were applied.

#### VBM

Voxel-wise comparisons of white matter volume in the patient and control groups were performed using an analysis of covariance (ANCOVA) followed by post hoc, two-sample t-tests (between DS and HC1, NDS and HC1, and DS and NDS, respectively) in SPM8. The threshold for ANCOVA test was set at p < 0.001, this initial screening threshold was adopted to optimize sensitivity for small structural changes in patients. However, in order to control more stringently for multiple comparisons, a combined voxel-level p < 0.0001 and cluster-level p < 0.05 (FWE corrected) was adopted for post hoc, two-sample t-tests.

The analyses of data from the relatives groups (contrasts between DS_R and HC2, NDS_R and HC2, and DS_R and NDS_R) were also completed using ANCOVA and post hoc two-sample t-tests. However, anticipating less volume alteration in otherwise unaffected relatives, we adopted a somewhat less conservative statistical threshold of uncorrected p < 0.001 and cluster extent of 100 voxels for both ANCOVA and post hoc two-sample t-tests.

For all the voxel-wise comparisons of white matter volume, an explicit white matter mask constructed from all subjects (n = 211) was applied to ensure that only voxels within the white matter were analyzed. Finally, whole brain volume (total gray and white matter volume; WBV) was used as covariate in every analysis. To further evaluate the potential impact of age we also specified a model with both WBV and age as nuisance variables. The pattern of results remained unchanged - not shown.

#### FA

The voxel-wise comparisons of skeletonised FA maps were performed using the *randomise*^62^ toolkit implemented in FSL, which carries out permutation-based, nonparametric inference. Comparisons of patients and relatives with their respective control groups were performed separately using ANCOVA and post hoc two-sample t-tests, with WBV as covariate. As in the analysis of volume, the threshold of the initial ANCOVA was set at an uncorrected P < 0.001. For comparisons involving the patient groups, we reported results at P < 0.05 corrected for multiple comparisons using the threshold-free cluster enhancement (TFCE) method^63^ in post hoc two-sample t-tests. For comparisons involving the relative groups we adopted a less stringent statistical threshold of uncorrected p < 0.001.

#### ROI analysis

To further characterize the functional significance of potential biological markers, clusters that showed selective volume or FA alterations in DS (or NDS) were defined as ROIs. The volume and FA values within each ROI were extracted from each patient’s dataset and correlations with PANSS scores (including total score, positive, negative and general psychopathological subscale scores, respectively), IQ and DUP were explored.

We also planned to examine any relationship between the volume and FA of ROI clusters showing volume or FA differences in patients.

The alpha value was accepted as p < 0.05 for ROI analyses.

## Additional Information

**How to cite this article**: Lei, W. *et al*. White matter alterations in first episode treatment-naïve patients with deficit schizophrenia: a combined VBM and DTI study. *Sci. Rep.* **5**, 12994; doi: 10.1038/srep12994 (2015).

## Supplementary Material

Supplementary Information

## Figures and Tables

**Table 1:** Demographic and clinical features.

	NDS (n = 42)	DS (n = 33)	HC1 (n = 41)	NDS_R (n = 37)	DS_R (n = 21)	HC2 (n = 37)
Female/Male	17/25	11/22	17/24	21/16	10/11	19/18
Age	23.38 ± 7.08	22.33 ± 6.90	23.49 ± 7.33	42.76 ± 8.13	43.00 ± 8.23	43.13 ± 9.50
tWMV	0.431 ± 0.05	0.420 ± 0.06	0.435 ± 0.04	0.432 ± 0.04	0.428 ± 0.05	0.441 ± 0.06
WBV	1.173 ± 0.12	1.175 ± 0.13	1.173 ± 0.09	1.112 ± 0.09	1.130 ± 0.11	1.144 ± 0.12
Education years	12.31 ± 2.46	11.45 ± 2.71	12.66 ± 2.42	9.89 ± 3.79	9.24 ± 3.71	10.35 ± 4.80
IQ	98.32 ± 13.49	95.95 ± 18.18	119.25 ± 12.89*	95.48 ± 15.25	98.43 ± 16.76	105.34 ± 15.52*
DUP (months)	5.71 ± 6.93	12.22 ± 12.04*				
Age of Onset	22.73 ± 7.14	20.41 ± 6.74				
PANSS-T	87.83 ± 16.40	97.64 ± 17.55*				
PANSS-P	25.36 ± 6.21	22.48 ± 7.62				
PANSS-N	16.52 ± 5.87	27.15 ± 7.82*				
PANSS-G	45.95 ± 9.22	48.00 ± 9.33				

Note: Demographic data are shown as mean ± standard deviation; *p < 0.05; DS, deficit schizophrenia; NDS, non-deficit schizophrenia; HC1, healthy controls age-and sex-matched with DS and NDS; DS_R, first degree relatives of DS patients; NDS_R first degree relatives of NDS patients; HC2, healthy controls age- and sex-matched with DS_R and NDS_R; tWMV, total white matter volume; WBV, whole brain volume = total gray matter volume + total white matter volume; DUP, duration of untreated psychosis; PANSS-T, PANSS total scores; PANSS-P, PANSS positive symptoms subscale scores; PANSS-N, PANSS negative symptoms subscale scores; PANSS-G, PANSS general psychopathological symptoms subscale scores.

**Table 2:** White matter volume differences between groups.

Regions	Cluster P value (FWE corrected)	Voxels	Peak Z value	MNI Coordinates (x, y, z)		
Analyses of patient groups						
DS > HC1						
Precentral Gyrus	0.013	774	5.88	20	−19	64
Precentral Gyrus	0.036	501	5.69	−26	−17	60
HC1 > DS						
Cerebellum Posterior Lobe	0.020	659	5.76	−1	−56	−17
Extra-Nuclear	0.000	4053	5.24	−14	−16	15
Extra-Nuclear	0.000	5980	5.09	34	−17	12
NDS > DS						
Extra-Nuclear	0.021	640	4.50	−22	−4	2
Extra-Nuclear	0.015	730	4.37	16	−1	−0
Analyses of relatives						
HC2 > DS_R						
Insula	0.662	105	3.57	34	−18	13
NDS_R > DS_R						
Extra-Nuclear	0.453	515	4.00	15	−14	8

Note: Analyses of patient groups were thresholded at voxel level p < 0.0001 & cluster level corrected (FWE < 0.05); Analyses of relatives were thresholded at voxel level p < 0.001 & cluster size >100 voxles.

**Table 3:** Fractional anisotropy differences between groups.

Regions	P value	Voxels	MNI Coordinates (x, y, z)		
Analyses of patient groups					
DS < HC1					
CC extending to SLF	0.006	5984	21	−40	43
CC extending to SLF	0.006	4117	−18	−44	38
Uncinate fasciculus	0.046	140	28	34	4
NDS < HC1					
CC extending to SLF	0.026	1131	20	−39	41
SLF extending to Posterior Cingulum	0.035	400	−14	−60	30
CC	0.031	262	−21	−39	41
SLF	0.040	159	35	−41	32
Analyses of relatives					
NO GROUP DIFFERENCES					

Note: Analyses of patient groups were thresholded at p < 0.05 TFCE corrected; Analyses of relatives were thresholded at p < 0.001 uncorrected; SLF, Superior Longitudinal Fasciculus; CC, corpus callosum.
