# Supplementary material for: White matter alterations in first episode treatment-naïve patients with deficit schizophrenia: a combined VBM and DTI study
Source: Sci Rep. 2015 Aug 10;5:12994. doi: 10.1038/srep12994 (PMC4530339; doi:10.1038/srep12994)
Supplement: Supplementary Information [file srep12994-s1.doc]

**White matter alterations in first episode treatment-naïve patients with deficit schizophrenia: a combined VBM and DTI study**

Wei Lei ^1¶^, Na Li ^1¶^, Wei Deng ^1^, Mingli Li ^1^, Chaohua Huang ^1^, Xiaohong Ma ^1^, Qiang Wang ^1^, Wanjun Guo ^1^, Yinfei Li ^1^, Lijun Jiang ^1^, Yi Zhou ^2^, Xun Hu ^3^, Grainne McAlonan ^4*^, Tao Li ^1*^

**SUPPLEMENTARY INFORMATION**

The DUP data was log-transformed to conform to assumptions of normality (Kolmogorov-Smirnov Z = 0.998, p = 0.272). Regression analysis with duration of untreated psychosis (DUP) as a predictor revealed no significant association between DUP and white matter volume. To further assess the effect of DUP on white matter volume, a median split of DUP data was used to divide the NDS group into two subgroups (long DUP group and short DUP group, each n = 21) and their white matter volume were compared (please see sample characteristics in Table S1). This analysis was performed in SPM8 as described in the main text. Patients with longer DUP had lower WMV than those with short DUP in regions including the frontoparietal region, middle temporal gyrus, cuneus and posterior lobe of the cerebellum (please see Table S2). However, there was no WMV difference in extra-nuclear regions between these groups, suggesting the lower extra-nuclear regional volumes found in DS cannot be explained by longer DUP.

**Table S1.** Demographic and clinical features for NDS patients with longer or shorter DUP

|  | **Long DUP**  **(n=21)** | **Short DUP**  **(n=21)** |
| --- | --- | --- |
| Female/Male | 7/14 | 10/11 |
| Age | 22.57±6.66 | 24.19±7.55 |
| tWMV | 0.427±0.04 | 0.435±0.06 |
| WBV | 1.174±0.13 | 1.172±0.11 |
| Education years | 12.33±2.35 | 12.29±2.63 |
| DUP (months) | 13.62±1.50 | 0.97±0.73* |
| Age of Onset | 21.44±6.62 | 24.11±7.55 |
| PANSS-T | 89.19±16.22 | 86.48±16.86 |
| PANSS-P | 25.38±5.07 | 25.33±7.30 |
| PANSS-N | 17.24±6.51 | 15.81±5.22 |
| PANSS-G | 46.57±9.22 | 45.33±9.41 |

Note: Demographic data are shown as mean ±standard deviation; *, p < 0.05;

Aberrations: NDS, non-deficit schizophrenia; Long DUP, NDS patients with longer DUP; Short DUP, NDS patients with shorter DUP; DUP, duration of untreated psychosis; tWMV, total white matter volume; WBV, whole brain volume= total gray matter volume + total white matter volume; PANSS-T, PANSS total scores; PANSS-P, PANSS positive symptoms subscale scores; PANSS-N, PANSS negative symptoms subscale scores; PANSS-G, PANSS general psychopathological symptoms subscale scores.

**Table S2．**White matter volume differences between patients with long or short DUP

| **Regions** | **Voxels** | **Peak Z value** | **MNI Coordinates**  **(x, y, z)** |
| --- | --- | --- | --- |
| **Short DUP > Long DUP** |  |  |  |
| Precentral Gyrus | 4247 | 4.11 | 32 -22 48 |
| Precentral Gyrus | 3160 | 3.54 | -32 -19 47 |
| Cingulate Gyrus | 721 | 3.4 | -11 18 34 |
| Middle Frontal Gyrus | 491 | 3.16 | 34 -8 46 |
| Cuneus | 330 | 3.01 | -19 -82 17 |
| Middle Temporal Gyrus | 306 | 2.82 | -34 -73 20 |
| Cerebellum Posterior Lobe | 372 | 2.71 | -1 -59 -23 |

Note: thresholded at p < 0.01 uncorrected & cluster extent ≥ 300 voxels.
